# Reviewer acknowledgment 2013

**DOI:** 10.1186/1880-6805-33-3

**Published:** 2014-02-05

**Authors:** Akira Yasukouchi

**Affiliations:** 1 Department of Physiological Anthropology, Faculty of Design, Kyushu University, 4-9-1, Shiobaru, Minami-ku, Fukuoka 815-8540, Japan

## Abstract

**Contributing reviewers:**

The editors of Journal of Physiological Anthropology would like to thank all our reviewers who have contributed to the journal in Volume 32 (2013).

## 

Chris Abbiss

Australia

Daijiro Abe

Japan

Vicent Balanza

Spain

Harold Bell

United States of America

Daniel K. Brown

United Kingdom

Chinmei Chou

Taiwan

Douglas E. Crews

United States of America

Sudip Datta Banik

Mexico

Cem Ekmekcioglu

Austria

Yuki Fujita

Japan

Katsuo Fujiwara

Japan

Yoshiyuki Fukuba

Japan

Yoshiyuki Fukuoka

Japan

Elena Godina

Russian Federation

Keita Hamasaki

Japan

Keiji Hayashi

Japan

Steven Heymsfield

United States of America

Shigekazu Higuchi

Japan

Kohji Hirakoba

Japan

Norio Hotta

Japan

Amelia Hubbard

United States of America

Tomoko Ichinose

Japan

Kaoru Inoue

Japan

Keita Ishibashi

Japan

Koichi Iwanaga

Japan

Masaharu Kagawa

Japan

Bibha Karmakar

India

Masatoshi Katagiri

Japan

Ryuichi Kawamoto

Japan

Yeon-Kyu Kim

Japan

Tetsuya Kimura

Japan

Shingo Kitamura

Japan

Eugene Kobyliansky

Israel

Narihiko Kondo

Japan

Takaaki Kondo

Japan

Kentaro Kotani

Japan

Katsuyasu Kouda

Japan

Tomoaki Kozaki

Japan

Jeong Beom Lee

Korea South

Xinxin Liu

Japan

Takahiro Maeda

Japan

Teodoro Marotta

Italy

Sarah Martin

United States of America

Joseph Maté

Australia

Ranjana Mehta

United States of America

Satoshi Muraki

Japan

Masashi Nakamura

Japan

Takayuki Nishimura

Japan

Nobuhiro Nishio

Japan

Hiroki Noguchi

China

Hiroyuki Nunome

Japan

Jun-ya Ohashi

Japan

Tetsuya Ohira

Japan

Hisato Ohno

Japan

Hidenobu Ohta

Japan

Kazushige Oshita

Japan

Stephane Perry

France

Yuka Saeki

Japan

Toshiyuki Saito

Japan

Yayoi Satsumoto

Japan

Lawrence Schell

United States of America

Yoshihiro Shimomura

Japan

Nami Someya

Japan

Koji Sugiyama

Japan

Yuji Takasaki

Japan

Yahiko Takeuchi

Japan

Jun Tayama

Japan

Stewart Thompson

United States of America

Craig Tokuno

Canada

Kumpei Tokuyama

Japan

Yuko Tsunetsugu

Japan

Hitoshi Wakabayashi

Japan

Jong Min Woo

Korea South

Tamaki Yamada

Japan

Miyo Yokota

United States of America

Shintaro Yokoyama

Japan

